# N2 and P3 responses in adolescents with non-suicidal self-injury and their association with clinical outcomes: a cohort follow-up study based on the affective stroop paradigm

**DOI:** 10.3389/fpsyt.2025.1564049

**Published:** 2025-06-24

**Authors:** Fei Yin, Feng Si, Wenlong Jiang, Shuhui Huo, Binquan Wang, Nan Yang, Jianqin Cao

**Affiliations:** ^1^ School of Nursing, Harin Medical University, Harin, China; ^2^ Key Laboratory of Human Factors and Ergonomics, State Administration for Market Regulation, China National Institute of Standardization, Beijing, China; ^3^ Psychiatry Department, The Third People’ Hospital of Daqing, Daqing, China

**Keywords:** non-suicidal self-injury, EEG, developmental outcomes, EEGNET, ERP, cognitive control, adolescent

## Abstract

**Background:**

Non-suicidal self-injury (NSSI), a prevalent psychiatric behavioral problem in adolescents, manifests diverse outcomes including cessation of remission, aggravation, and even progression to suicidal behaviors. It is crucial to track its progression and identify predictors of clinical outcomes in NSSI. The aim of this study was to determine whether the N2 and P3 responses in adolescents with NSSI were defective in the presence of the affective Stroop paradigm and associated with their clinical outcomes.

**Methods:**

Participants were selected from an ongoing longitudinal study designed to predict clinical outcomes of NSSI in adolescents. Twenty-six in the remission group (RG), twenty-nine in the aggravation group (AG), and twenty-seven in the healthy group (HG) completed the affective Stroop task with EEG. Accuracy and reaction times (RTs) served as behavioral indexes, while N2 and P3 amplitude served as electrophysiological indexes; they were analyzed across groups. We used the EEGNet model to predict the NSSI clinical outcomes with EEG component.

**Result:**

No significant main effects of group or affective stimuli or an effect of their interaction were observed on accuracy (*p* > 0.05). For RTs, there was a significant main effect of group, with slower RTs observed in the AG compared to the HG (*p* < 0.05). For N2 and P3 amplitude, there were significant main effect of group and affective stimuli and an effect of their interaction. Under neutral stimuli, the N2 amplitude in the AG was significantly larger than that in the RG (*p* < 0.05) and the HG (*p* < 0.01), while the P3 amplitude in AG was significantly smaller than that in the HG (*p* < 0.05), but there was no significant difference between RG and AG(*p* > 0.05). The EEGnet model demonstrated that N2 amplitudes elicited by neutral stimuli achieved the highest classification accuracy (92.31%) for predicting clinical outcomes of NSSI.

**Conclusion:**

The findings indicate that NSSI is linked to cognitive processing deficits, including impaired control and resource allocation to stimuli. Additionally, N2 amplitudes were shown to reliably predict clinical outcomes in NSSI.

## Background

Non-suicidal self-injury (NSSI) refers to deliberate and direct damage to body tissue without suicidal intent (1). While not intended to result in death, evidence suggests that NSSI can predict future suicide attempts and fatalities (2, 3). A comprehensive global meta-analysis of an extensive sample revealed that the lifetime prevalence of non-suicidal self-injury (NSSI) among adolescents is 22.1% (4), with evidence indicating a gradual upward trend. NSSI in this demographic serves as a significant predictor of suicide, substantially elevating the associated risk (5). According to the 2019 Global Burden of Disease data, NSSI ranks as the third leading cause of disability-adjusted life years (DALYs) globally among individuals aged 10–24 years (6). This condition imposes a considerable socio-economic burden and constitutes a critical public health issue that necessitates urgent attention and intervention. The classification of NSSI as an independent category in the DSM-5 underscores its significance and the necessity for further research.

NSSI typically begins in early adolescence and progresses into diverse developmental trajectories over time. The clinical outcome of NSSI in adolescents is usually characterized by several possible developmental paths: cessation of short-term episodes, repeated long-term episodes, and even progression to suicidal behaviors (7). A study involving 13- to 19-year-olds identified two patterns of NSSI frequency: Low Stable and High Persistent (8). Similarly, a year-long study of 3,381 adolescents found two trajectory groups: a remission group (87.4%) and an aggravation group (12.7%) (9). These longitudinal studies highlight the varied developmental pathways of NSSI, emphasizing the importance of tailored prevention and intervention strategies. While identifying trajectories is crucial, determining predictors of clinical outcomes holds even greater importance.

The clinical outcomes of adolescents engaging in NSSI exhibit significant individual variability, influenced by a multitude of factors. Early identification of potential clinical trajectories is crucial for psychiatric professionals, as it enables the timely and effective implementation of tailored treatment and intervention strategies, thereby mitigating the risk of adverse clinical outcomes. Current forecasting methods largely rely on validated scales. From the perspectives of family system theory and cumulative risk, family conflict risk has been identified as a key predictor of NSSI addiction (7). Other studies highlight the roles of interpersonal factors, such as unstable relationships and parental criticism, as predictors of high NSSI fluctuations (9). Personal factors, including impulsive behaviors, depression (9), emotion regulation difficulties, self-compassion deficits (10), psychiatric symptoms, and comorbidities (8), also contribute significantly. However, reliance on subjective self-reports introduces bias and reduces diagnostic accuracy. Objective measurements based on individual characteristics offer a more reliable alternative, presenting a promising avenue for predicting NSSI outcomes with greater precision.

Behavioral findings from various cognitive tasks have provided insights into cognitive control in subgroups of individuals with NSSI. Frequent NSSI has been associated with poorer performance on the Criticism Gambling Task, suggesting that impulsive decision-making in response to criticism may serve as a critical indicator of NSSI progression (11). Additionally, high-severity NSSI groups have been found to exhibit working memory deficits, whereas low-severity groups demonstrate impaired inhibitory control (12). Electroencephalogram (EEG) devices allow for the collection of stimulus-evoked EEGs, or event-related potentials (ERPs), through specifically designed experimental tasks. Previous studies have reported that adolescents with NSSI, compared to HC, exhibit increased N2 amplitudes (13) and decreased P3 amplitudes (14), indicating significant cognitive control dysfunction. Further, Zhao et al. identified that adolescents with NSSI displayed smaller N2 and larger P3 responses during negative emotional face stimuli (15). However, no ERP-based studies currently address clinical outcomes of NSSI, highlighting the potential of investigating neurocognitive factors using ERP methodologies.

ERPs offer high temporal resolution, making them valuable for studying cognitive processes with millisecond precision (16, 17). The N2 component, located in the anterior central brain region, is indicative of conflict processing, with its amplitude reflecting the degree of attention control (18). The P3 component is thought to reflect the individual’s attention or alertness, allocation of attention resources and attention transfer (19). Cognitive processing involves the activation and coordination of multiple brain regions. For instance, the affective Stroop task studied via fMRI has demonstrated activation in regions such as the amygdala, insula, and prefrontal cortex, alongside cognitive networks involving the prefrontal cortex, superior temporal lobe, and insula (20). This study seeks to examine the cognitive processing features of NSSI outcome groups using an affective Stroop paradigm, which is a reliable tool for assessing cognitive functions (21). Given that negative emotions can impair cognitive performance (22, 23), the affective Stroop task is a classical paradigm used in recent years (24, 25). Washburn et al. noted that individuals frequently engaging in NSSI might have a behavioral addiction (26), sharing neurobiological roots with addiction (27). Drug addiction is marked by cue reactivity (28), shown through behavioral and neurobiological methods like ERP. A prior study indicated that adolescents with NSSI might show altered behavioral and brain responses to NSSI cues (29). Therefore, a novel affective Stroop paradigm which contains neutral and NSSI images as stimuli was adapted to evaluate the effects of emotional valence on cognitive tasks across NSSI outcome groups. We used the affective Stroop paradigm to assess how emotional distraction from NSSI-related images impacts cognitive control, as reflected in Stroop performance and ERPs. We hypothesize that adolescents with worsening NSSI would show larger N2 and smaller P3 amplitudes than those in remission or healthy controls. The objective of this study was to identify sensitive electrophysiological markers capable of predicting various clinical outcomes of NSSI, with the intent of offering objective screening tools and therapeutic targets for clinical psychiatric practitioners.

Deep learning has demonstrated significant capability in automatically learning and extracting features from raw data, making it highly applicable to EEG signal analysis (30, 31). Compared to conventional methods, deep learning techniques offer several advantages, including robust feature extraction and representation, end-to-end training, and superior generalization. Among these, EEGNet, a convolutional neural network (CNN) specifically designed for Brain-Computer Interface (BCI) applications (32), has shown exceptional performance in processing task-related EEG datasets. EEGNet is designed for processing EEG signals. It addresses the complexities of non-stationary EEG data with a carefully structured architecture. The model starts with a temporal convolution layer to identify temporal patterns, then uses depthwise convolution to learn spatial filters for each channel, and concludes with separable convolution to combine these filters and extract additional features. These include applications such as P3 visual-evoked potentials (33), movement-related cortical potentials (MRCP) (34), and sensory motor rhythms (SMR) (35). In this study, ERP components such as N2 and P3 were extracted from the affective Stroop dataset for comparative analysis using EEGNet. The secondary objective was to evaluate the accuracy of clinical outcome predictions for NSSI by using these ERP components as input features to the EEGNet model.

## Methods

### Participants

Our previous study tracked the trajectories of NSSI in 550 adolescents with NSSI behavior (12–18 years old) across three waves of follow-up a one-year period, each separated by a six-month interval. A total of 502 adolescents completed the entire follow - up survey, and 48 adolescents were lost to follow-up. We tested for differences in gender, age, and baseline NSSI between participants lost to follow-up and those effectively tracked. Results showed no significant differences (*p* < 0.05), suggesting random sample loss and no impact on research outcomes. All the research subjects in this study were from the community and had no specific disease diagnoses. NSSI was evaluated based on DSM-5 criteria (36) for the past six months, which includes eight specific behaviors: cutting/stabbing, hair pulling, head banging, biting, cauterizing, scratching, hitting, and rubbing until bleeding occurred. The adolescents rated their engagement in each NSSI behavior for the past six-month on a 4-point Likert-type scale (1 = “never,” 2 = “once or twice,” 3 = “three to five times,” and 4 = “six times or more”). If one or more NSSI behaviors were reported, it was classified as an instance of NSSI behavior. Latent class analysis revealed two subgroups remission group (RG) (n = 371, 73.90%), and aggravation group (AG) (n = 131, 26.10%). In RG, participants showed a decreased frequency or medical severity of NSSI episodes from the start to the end of the follow-up. In AG, participants showed an increased frequency or medical severity of NSSI episodes from the start to the end of the follow-up. Participants in RG and AG were recruited from our previous longitudinal study designed to predict clinical outcomes of NSSI in adolescents. The inclusion criteria: φAdolescents met the DSM-5 diagnostic criteria for NSSI, with NSSI occurring at least once in the past year; κNormal visual acuity or corrected visual acuity; ΛRight-handed. Exclusion criteria: φThose with suicidal ideation or behavior in the past year with a structured interview; κPatients with other mental disorders; ΛPatients with a history of traumatic brain injury, epilepsy, etc; μThose who have received drug or psychotherapy in the past year. Healthy group (HG) were healthy adolescents recruited through advertisements. The inclusion criteria: φNo past or current mental disorders as per the Brief International Neuropsychiatric Interview for Children and Adolescents (ICAO-5); κNo history of NSSI behaviors; ΛNormal or corrected visual acuity; μRight-handed. Exclusion criteria: the same as the NSSI group.

Those who met these criteria underwent structured clinical interviews based on DSM-5 criteria conducted by counselors for group assignment confirmation. Participants volunteered for the study, provided informed consent, could withdraw at any time, and were compensated with cash. Ethical approval was obtained from the Ethics Committee of Harbin Medical University Daqing Campus, and the study adhered to the ethical standards outlined in the World Medical Association Declaration of Helsinki.

### Task paradigm

**Figure 1:**
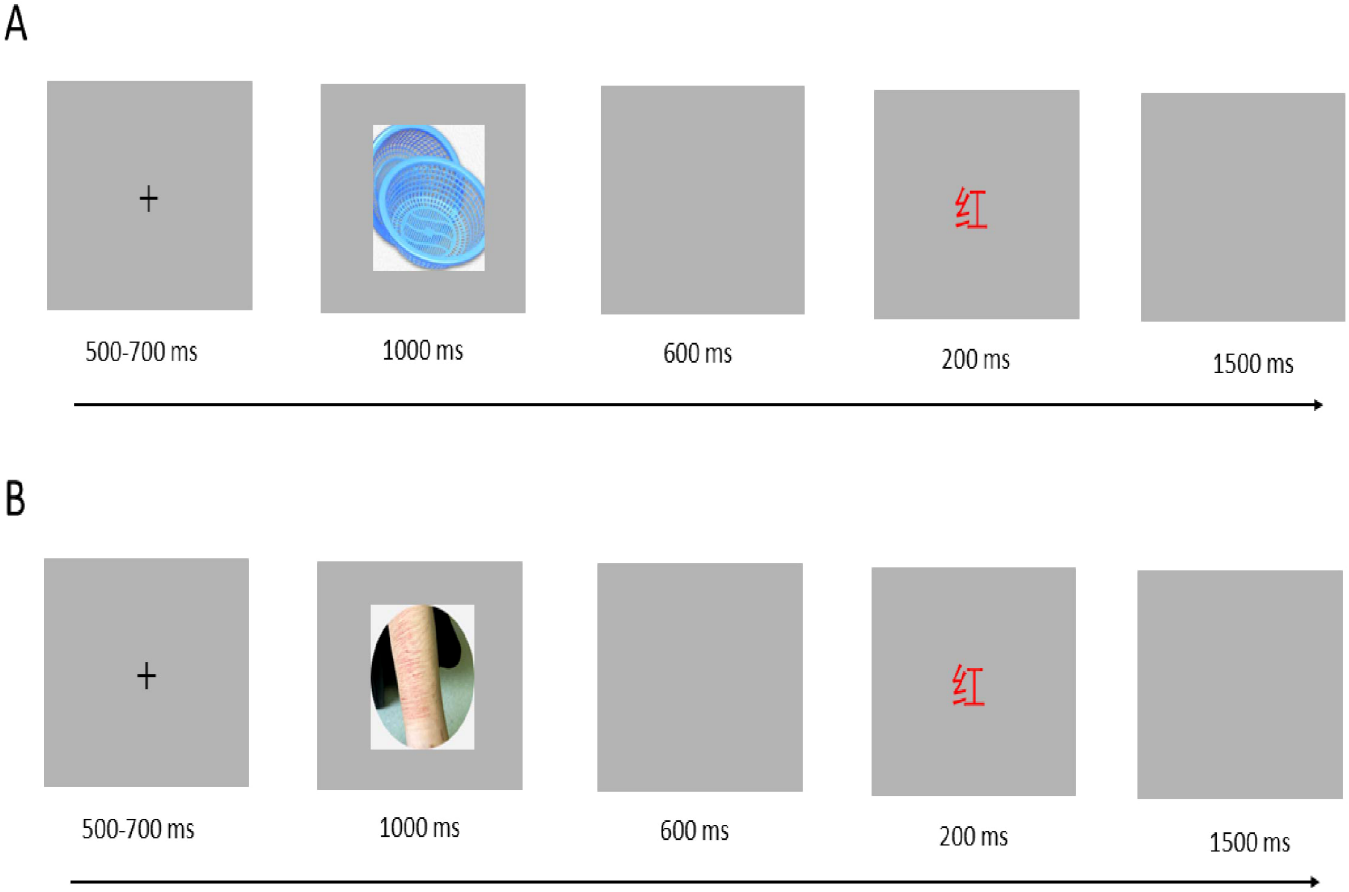
Experiment paradigm. **(A)** Neutral trial; **(B)** Negative trial.

All participants were required to complete the affective Stroop task during EEG recording. The task was created using E-prime 3.0 (see **Figure 1**). It consisted of two blocks, each containing 120 neutral trials and 120 negative trials. The trials were presented in a pseudo-randomized order within each block. The emotional stimuli included 30 non-NSSI-related neutral pictures selected from the Chinese Affective Picture System (CAPS) (37, 38) and 30 neutral pictures sourced from NSSI images of the participants. We gave participants a detailed NSSI image-selection guideline. It required images to clearly depict study-relevant NSSI acts, restricting behaviors to common ones (e.g., cutting, burning, scratching). We also asked them to avoid overly distracting or complex images for consistent core content across participants. Prior to the main experiment, we pre-tested participant-specific NSSI images with a demographically similar volunteer group. Images were rated on a standardized arousal scale, and those with extreme ratings were excluded. We then selected images for each participant within a narrow arousal range to standardize stimulus arousal.

The two sets of emotional pictures differed significantly in valence [F _(1, 58)_ = 136.51, P < 0.001] but were similar in arousal [F _(1, 58)_ = 5.17, P = 0.21]. The literal stimuli consisted of single words presented in one of four colors (red, yellow, blue, or green). Two experimental conditions were used: the congruent condition, where each color word was displayed in its corresponding color, and the incongruent condition, where each color word was presented in a color other than the one it referred to (e.g., the word “red” displayed in yellow).

Each trial began with a white “+” displayed for 500–700 ms in the center of the screen on a grey background, followed by an emotional picture presented for 1000 ms, then a blank screen for 600 ms, followed by a color word displayed for 200 ms, and a final blank screen for 1500 ms. Participants were required to determine whether the word and color were congruent. If they were congruent, participants pressed the “D” key with their left index finger; if they were incongruent, participants pressed the “J” key with their right index finger. Both response time and accuracy were recorded in this study.

### EEG recording and processing

The EEG data were recorded using a Neuroscan ESI 64-channel system, equipped with an Ag/AgCl electrode cap and the extended international 10–20 electrode placement. The system was configured with a bandpass filter of 0.05–100 Hz and a sampling rate of 500 Hz. A left mastoid electrode was used as the online reference, which was later re-referenced offline to the common average (39). Vertical electrooculogram (EOG) was recorded from electrodes placed above and below the left eye, while horizontal EOG was captured from electrodes positioned at the outer canthi. Electrode impedance was kept below 5 kΩ throughout the task.

Experimental conditions, equipment, and physiological responses can introduce interference that affects the collected signals. To minimize these effects, preprocessing steps were applied to the EEG data. EOG blink artifacts were corrected using linear regression estimation (40). The EEG signals were band-pass filtered between 0.05 Hz and 30 Hz and segmented into 1000 ms epochs, with a 200 ms pre-stimulus baseline. Specifically, we used an epoch time window from –200 ms pre-stimulus to 1000 ms post-stimulus. Epochs with voltage deviations exceeding ±80 μV were excluded from the ERP analysis. On average, the retained neutral trials in HG, RG, and AG groups were 103 ± 14, 94 ± 21, and 93 ± 19 respectively, with no significant inter-group difference (F =2.496; *p* = 0.089). The retained negative trials in HG, RG, and AG groups were 96 ± 20, 91 ± 17, and 86 ± 22 respectively, with no significant inter-group difference (F =1.771; *p* = 0.175). Separate averages for the congruent and incongruent conditions were computed for neutral and negative stimuli, respectively. Incorrect responses may be associated with different cognitive processes, such as error detection and correction, which could confound the analysis of the N2 and P3 components. Only trials with correct responses were included in the calculation of average ERPs.

### ERP analysis

Based on ERP components linked to cognition as reported in previous research (41–43), as well as the characteristics of the ERP waveforms observed in the current study, specific ERP components were selected for further analysis. For N2 analysis, frontal- central electrodes were selected. These regions are related to cognitive processes like conflict monitoring and error detection, relevant to our study. The P3 component is thought to reflect widely - distributed attentional and evaluative processes in the frontal network. The amplitudes of the N2 (260–320 ms; FZ, FCZ, PZ, CPZ, PZ) and P3 (320–370 ms; F3, F1, FZ, F2, F4, FC3, FC1, FCZ, FC2, FC4) components were examined. For each ERP component, the mean amplitude within the specified time window was calculated. Our analyses were based on the raw ERP amplitudes for each condition.

### Statistical analysis

A repeated-measures analysis of variance (ANOVA) was conducted to assess accuracy, reaction time (RT), and ERP component amplitude across different subgroups. The analysis included a three-way repeated-measures design with group (healthy, declining, and worsening) as the between-subject factor, and emotion (neutral, negative) and congruency (congruent, incongruent) as within-subject factors. Statistical analyses were performed using repeated-measures ANOVAs with Greenhouse-Geisser corrections at a significance level of 0.05. Significant interaction effects were followed by simple effect analysis, with pairwise comparisons adjusted using the Bonferroni method and an alpha value of 0.05 divided by the number of comparisons.

In the model, a 64-channel EEG signal was used as the input, and the group served as the output. The model was trained and tested through a deep convolutional layer followed by a deep separable convolutional layer. This network extracts intrinsic characteristic information from the waveform using convolutional kernels, enabling the prediction of clinical outcomes associated with NSSI. We inputted raw EEG time series data from the Stroop task into the EEGNet model, which includes ERP components like N2 and P3 amplitudes. This allows the model to autonomously identify relevant features and patterns. The datasets were split in two ways: randomly into three subsets-60% for training, 20% for validation, and 20% for testing-ensuring that no participant’s data appeared in more than one subset, and employing 5-fold cross-validation. We added dropout layers to our EEGNet architecture to enhance model robustness and prevent overfitting by randomly setting a portion of input units to zero during training.

## Results

### General characteristics

Ninety adolescents were recruited from the longitudinal study, with 30 participants in each subgroup (HG, RG, AG). Eight participants were excluded: two for accuracy below 75%, three for z-scores above 3.29, and three due to excessive EEG artifacts in over 25% of trials. The final sample included 82 participants (mean age 15.69 ± 1.73; 49 females): 27 in HG, 26 in RG, and 29 in AG. Using G*Power 3.1 with effect size f=0.25f=0.25, α=0.05α=0.05, and 1−β=0.951−β=0.95, the calculated total sample size was 68, with 23 per group to ensure result robustness. The demographic characteristics of all participants are summarized in **Table 1**. There were no significant differences in general demographic data among the three groups of participants.

**Table 1:** Participant characteristics of the HG, RG, and AG.

Variable	HG (n=27) RG (n=26) AG(n=29)			Test statistic
Age	16.17 ± 2.19	15.43 ± 1.51	15.26 ± 1.81	F_(2, 52)_ = 2.73,
Sex				*p* = 0.251
Male	12	10	11	*x* ^2^ = 0.297.
Female	15	16	18	*p* = 0.862
Family economic status				*x* ^2^ = 3.694,
Rich	8	9	7	*p* = 0.449
Medium level	13	7	14	
Poor	6	10	8	
Father’s education				*x* ^2^ = 4.563,
Junior high school and below	2	4	8	*p* = 0.601
High school and technical secondary school	15	13	14	
Junior college	7	7	5	
Undergraduate Graduate and above	3	2	2	
Mather’s education				*x* ^2^ = 9.031,
Junior high school and below	3	7	8	*p* = 0.172
High school and technical secondary school	8	12	13	
Junior college	11	5	4	
Undergraduate Graduate and above	5	2	4	
Family Type				*x* ^2^ = 3.056,
Complete family	23	18	19	*p* = 0.217
Broken family	4	8	10	

### Behavioral performance

**Table 2** presents the accuracy and reaction times (RTs) for the different groups.

**Table 2:** The accuracies and average reaction times in the different groups (M ± SD).

Variable	Group	Neutral stimuli		Negative stimuli	
		Congruent	Incongruent	Congruent	Incongruent
Accuracies	HG	0.94 ± 0.06	0.84 ± 0.05	0.94 ± 0.05	0.81 ± 0.04
	RG	0.94 ± 0.07	0.84 ± 0.09	0.94 ± 0.11	0.79 ± 0.02
	AG	0.95 ± 0.05	0.84 ± 0.06	0.94 ± 0.07	0.80 ± 0.06
Reaction time (ms)	HG	609 ± 91	646 ± 101	599 ± 113	624 ± 106
	RG	623 ± 111	659 ± 109	606 ± 112	610 ± 122
	AG	666 ± 91	712 ± 97	681 ± 147	714 ± 141

**Figure 2:**
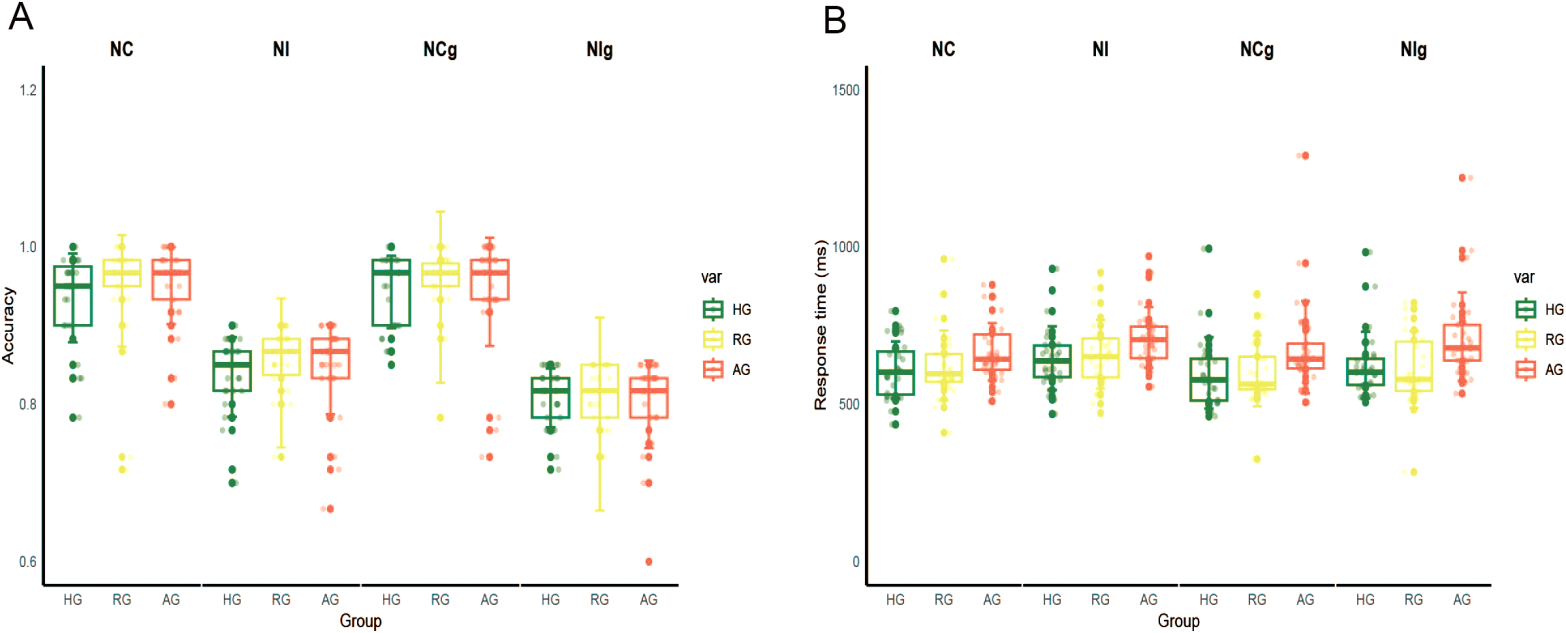
The results of behavior data. **(A)** The accuracy in the HG, RG and AG. **(B)** The response time in the HG, RG and AG. NC, congruent in neutral stimuli; NI, incongruent in neutral stimuli; NCg, congruent in negative stimuli; NIg, incongruent in negative stimuli.

The ANOVA of accuracy revealed a significant main effect of emotion type (F _(1,79)_ = 21.494, *p* < 0.001, η^2^ = 0.214), indicating that accuracy was higher for neutral stimuli compared to negative stimuli (*p* < 0.001). The main effect of congruency was also significant (F _(1, 79)_ = 682.573, *p* < 0.001, η^2^ = 0.898), showing that accuracy was higher for the congruent condition than for the incongruent condition (*p* < 0.001). The main effect of group on accuracies was not significant (F _(1, 79)_ = 0.096, *p* = 0.909, η^2^ = 0.002) (**Figure 2A**).

The ANOVA of RTs showed a significant main effect of group (F _(2,79)_ = 4.145, *p* < 0.05, η^2^ = 0.095), with slower RTs observed in the AG compared to the HG (*p* < 0.05). The main effect of congruency was also significant (F _(1, 79)_ = 65.734, p < 0.001, η^2^ = 0.454), indicating that RTs were slower for the incongruent condition compared to the congruent condition (p < 0.001) (**Figure 2B**).

### ERP results

**Table 3** presents the average amplitude for each ERP component in the different groups.

**Table 3:** The average amplitude for each ERP component in the different groups (M ± SD).

Component	Group	Neutral stimuli		Negative stimuli	
		Congruent	Incongruent	Congruent	Incongruent
N2	HG	1.09 ± 0.83	-0.41 ± 0.86	-0.41 ± 0.83	-0.27 ± .81
	RG	-2.16 ± 0.85	-1.66 ± 0.88	-3.70 ± 0.85	-3.63 ± 0.83
	AG	-3.84 ± 0.80	-3.98 ± 0.83	-3.30 ± 0.80	-2.89 ± 0.78
P3	HG	4.12 ± 0.75	2.10 ± 0.82	2.07 ± 0.78	1.39 ± 0.76
	RG	1.05 ± 0.77	0.19 ± .83	-1.98 ± 0.79	-2.87 ± 0.78
	AG	-0.25 ± 0.73	-1.20 ± 0.79	-1.43 ± 0.75	-1.44 ± 0.74

### N2 amplitude

**Figure 3:**
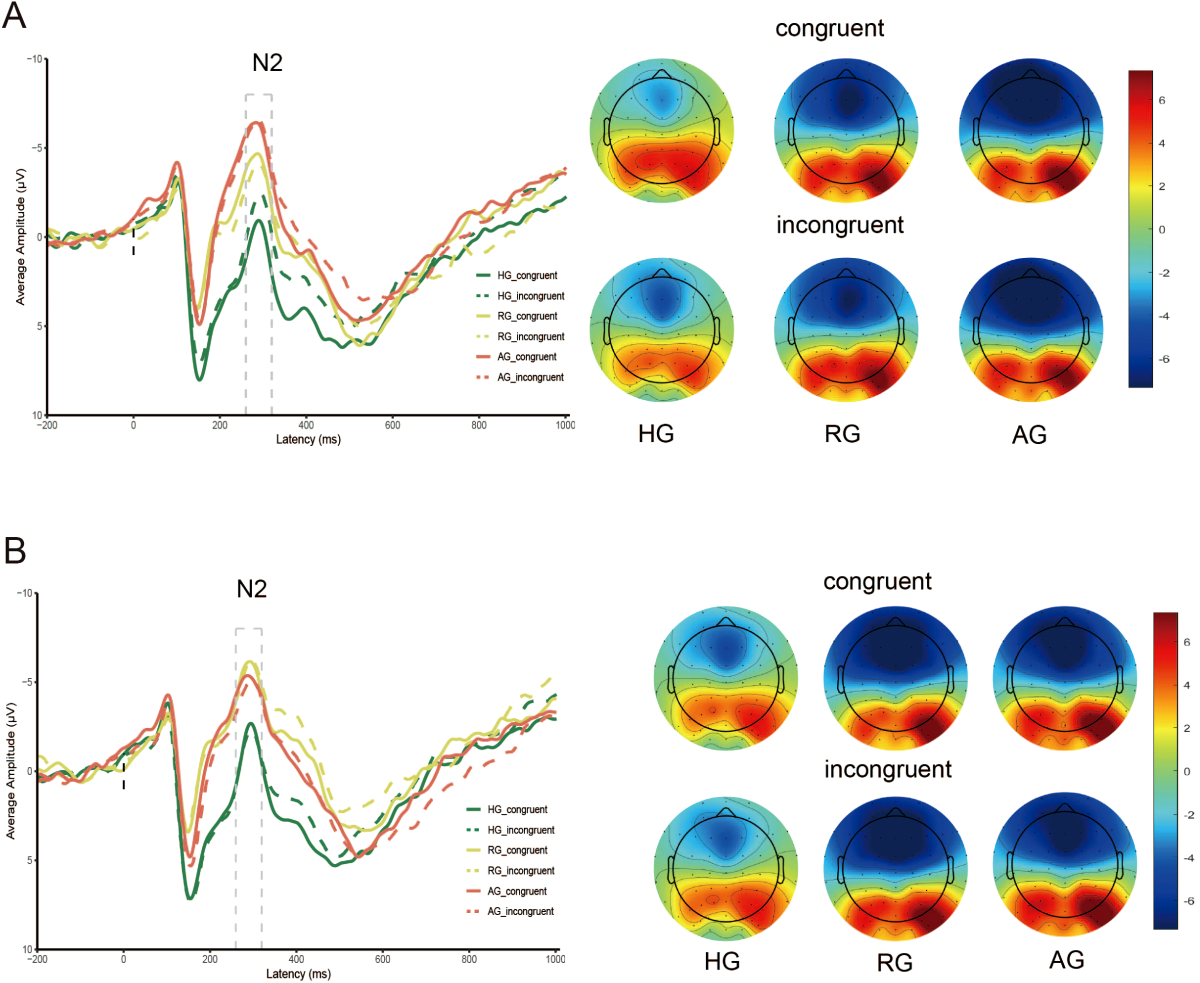
N2 amplitude evoked by HG, RG and AG. **(A)** neutral stimuli; **(B)** negative stimuli.

The results, presented in **Table 4** and **Figure 3**, revealed a significant main effect of emotion type (F _(1, 79)_ = 7.271, *p* < 0.01, *η* ^2^ = 0.084), where the N2 elicited by negative stimuli was larger than that elicited by neutral stimuli. The main effect of group was also significant (F _(2, 79)_ = 7.091, *p* < 0.01, η^2^ = 0.152). Specifically, the N2 in the HG was significantly smaller than in the RG and AG (*ps* < 0.05). However, there was no significant difference between RG and AG (*p* = 0.089). The interaction between group and emotion was significant (F _(2, 79)_ = 16.934, *p* < 0.001, η^2^ = 0.300). Under neutral stimuli, the N2 in HG was significantly smaller than in the RG (*p* < 0.05) and AG (*p* < 0.01), with the RG also being significantly smaller than the AG (*p* < 0.05). Under negative stimuli, the N2 in HG was significantly smaller than in the RG (*p* < 0.01) and AG (*p* < 0.05), and there was no significant difference between RG and AG (*p* = 0.101).

**Table 4:** Results from repeated measures ANOVA for N2 amplitudes (μV).

Effect	F	*p*	η^2^
Emotion type	7.271	0.009	0.084
Congruency	0.389	0.535	0.005
Group	7.091	0.001	0.152
Emotion × Congruency	3.109	0.082	0.038
Emotion × Group	16.934	<0.001	0.300
Congruency × Group	2.281	0.109	0.055
EmotionÍ Congruency × Group	4.068	0.021	0.093

The interaction between group, emotion, and congruency was significant (F _(2, 79)_ = 4.068, *p* < 0.05, η^2^ = 0.093). Under neutral stimuli, the N2 in HG was smaller than in RG (*p* < 0.01) and AG (*p* < 0.001) for both congruent and incongruent conditions, and the N2 in RG was also smaller than in AG for the congruent condition (*p* < 0.01). Under negative stimuli, the N2 in HG was smaller than in RG (*p* < 0.001) and AG (*p* < 0.01) for both congruent and incongruent conditions, and there was no significant difference between RG and AG for both congruent and incongruent conditions (*p* = 0.199; *p* = 0.084).

### P3 amplitude

**Figure 4:**
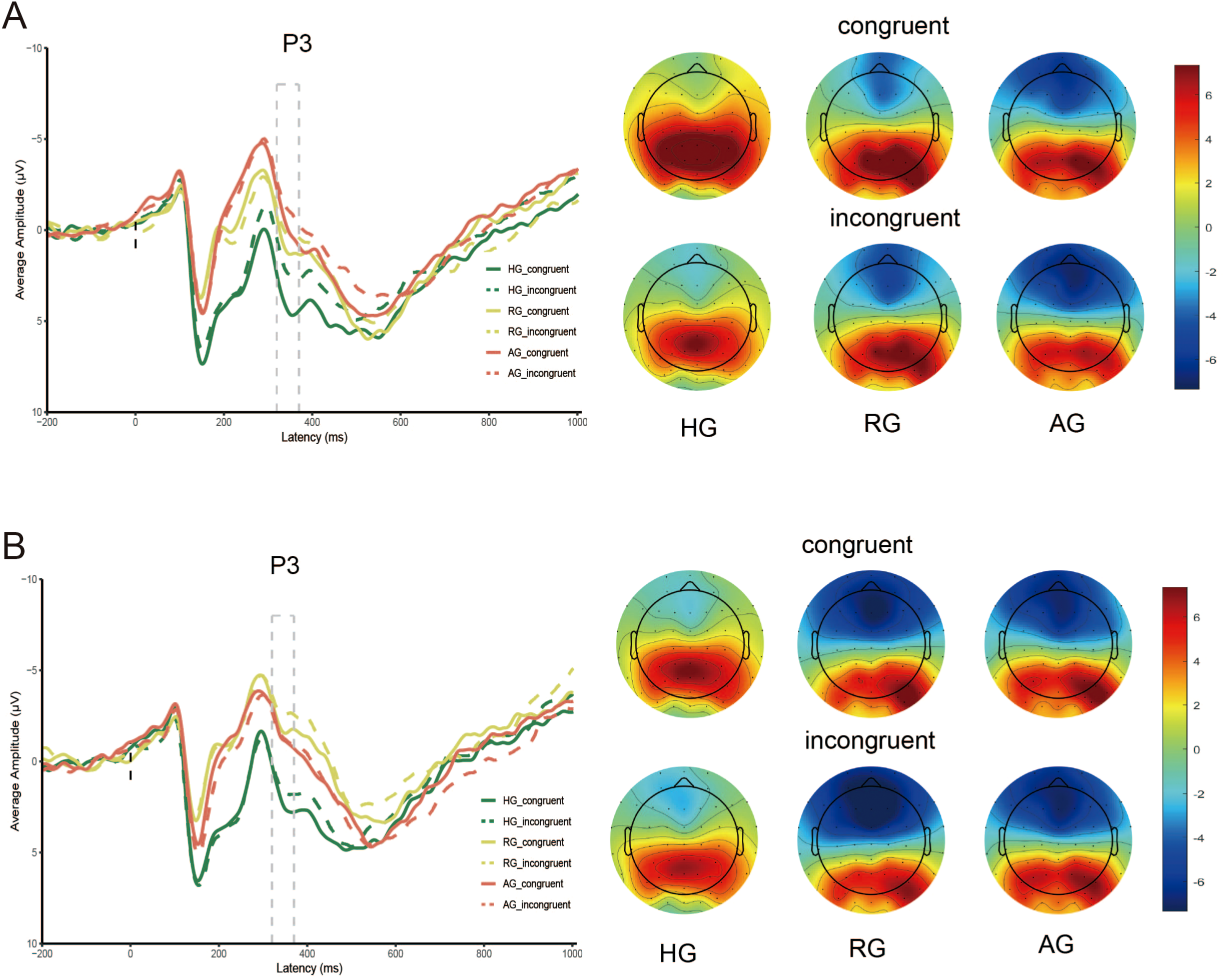
P3 amplitude evoked by HG, RG and AG. **(A)** neutral stimuli; **(B)** negative stimuli.

As shown in **Table 5** and **Figure 4**, the main effect of emotion type was significant (F _(1, 79)_ = 73.858, *p* < 0.001, η^2^ = 0.483), with the P3 elicited by neutral stimuli being larger than that elicited by negative stimuli. The main effect of group was also significant (F _(2, 79)_ = 7.607, *p* < 0.001, η^2^ = 0.161), where the P3 in HG was significantly larger than in RG (p < 0.01) and AG (p < 0.01). However, there was no significant difference between RG and AG (*p* = 0.158). The interaction between group and emotion was significant (F (_2, 79)_ = 12.029, *p* < 0.001, η^2^ = 0.233). Under neutral stimuli, the P3 in HG was significantly larger than in the AG (*p* < 0.01), but there was no significant difference between RG and AG (*p* = 0.053) and between HG and RG (*p* = 0.066). Under negative stimuli, the P3 in HG was significantly larger than in the RG (*p* < 0.001), but there was no significant difference between RG and AG (*p* = 0.101) and HG and AG (*p* = 0.128).

**Table 5:** Results from repeated measures ANOVA for P3 amplitudes (μV).

Effect	F	*p*	η^2^
Emotion type	73.858	<0.001	0.483
Congruency	15.609	<0.001	0.165
Group	7.607	<0.001	0.161
Emotion × Congruency	4.978	0.029	0.059
Emotion × Group	12.029	<0.001	0.483
Congruency × Group	1.222	0.300	0.030
EmotionÍ Congruency × Group	1.411	0.250	0.034

The main effect of congruency was significant (F _(1, 79)_ = 15.609, *p* < 0.001, η^2^ = 0.165), with the P3 elicited by the congruent condition being significantly larger than that elicited by the incongruent condition (*p* < 0.001).

No significant interaction effect between group, emotion, and congruency was observed (F _(2, 79)_ = 1.411, *p* = 0.250, η^2^ = 0.034). Simple effects analysis revealed that, under neutral stimuli, the P3 elicited by the congruent condition in HG was larger than in AG (*p* < 0.001). Under negative stimuli, the P3 in HG were larger than in RG (*ps* < 0.01) for both congruent and incongruent conditions.

### Prediction results on the EEGNet

We identified N2 and P3 components during neutral and negative stimulation using the EEGNet model to predict NSSI clinical outcomes. Under neutral stimuli, the ERP components achieved peak accuracies of 92.31% for N2 and 76.60% for P3, while under negative stimuli, the accuracies were 71.16% for N2 and 49.57% for P3. These results indicate that N2 performs best under neutral stimuli, with all components showing reduced accuracy under negative stimuli.

## Discussion

This study explored cognitive control in adolescents with varying NSSI clinical outcomes using an affective Stroop task, supporting the role of cognitive control as a predictor of NSSI and its clinical outcomes. Specifically, compared with the healthy group, NSSI groups had higher RTs, N2 and P3 amplitude, indicating that attention control and attention resource allocation in adolescents with NSSI were impaired. Following the introduction of emotional stimuli, the N2 amplitude demonstrated a significant impact in forecasting the clinical outcomes of NSSI. The N2 elicited by neutral stimuli exhibited significant differences between the RG and AG groups, with the AG displaying a larger N2 response than the RG. This suggests that the N2 component serves as a more sensitive indicator for distinguishing the clinical outcomes of NSSI.

Our research utilized neutral and negative images with distinct valence differences as stimuli in the affective Stroop task. The results indicated that negative stimuli impaired cognitive performance relative to neutral stimuli, leading to slower RTs and lower accuracy. This suggests that negative emotions should be closely monitored in individuals with NSSI to prevent adverse outcomes related to impaired cognitive response (44). The RG and AG groups exhibited slower RTs compared to the HG, suggesting lower processing efficiency and impaired cognitive control. Participants with required more time to perform conflict detection and task-related activities. No significant differences in accuracy were observed between groups, indicating that NSSI primarily affected processing efficiency rather than task effectiveness, although this finding requires further confirmation due to the small sample size.

Negative stimuli evoked greater N2 amplitudes than neutral stimuli, demonstrating that negative stimuli can rapidly and automatically capture attention and utilize attentional resources. Yuan et al. reported that individuals display heightened sensitivity to negative stimuli, processing them at an earlier stage (45). The results showed a significant difference in N2 amplitudes among the HG, RG, and AG, with the amplitude exhibiting a gradient change pattern across these groups. These findings indicate that participants in RG and AG, particularly AG, exhibit abnormalities in cognitive control mechanisms. Hanslmayr et al. observed that individuals responded to increased conflict levels by temporarily enhancing cortical responses to task-relevant information, which improved attention control performance instead of suppressing task-irrelevant information (46). Participants in RG and AG were not required to inhibit or overcome interference caused by affective stimuli. This absence of inhibition enhanced attention to target stimuli, resulting in larger N2 components. In contrast, HG displayed superior attention control, allowing effective inhibition of task-irrelevant information and successful interference management during task completion (47). Under neutral stimuli, participants in AG exhibited more pronounced N2 amplitude deficits compared to RG, with significant deficits in incongruent N2 amplitude relative to HG. While negative stimuli evoke strong emotions and neural variability, neutral stimuli may better predict emotion-independent cognitive control. The N2 amplitude differences in RG and AG under neutral stimuli likely indicate a baseline cognitive control deficit, unaffected by emotional arousal. Our findings suggest that N2 amplitude variations can distinguish between RG and AG, highlighting differences in non-emotional conflict management. Further research is needed to explore cognitive control deficits and their effects on the groups’ functioning. A larger N2 amplitude generally indicates heightened conflict monitoring or greater cognitive- control difficulty under emotional interference. In our study, AG adolescents might have hyper-reactive conflict detection. Their brains rapidly detect conflicts upon emotional stimulation, resulting in a larger N2, implying more effort in conflict monitoring than others. These results suggest that N2 amplitude serves as a sensitive marker for distinguishing HG from NSSI groups, as well as between RG and AG, providing an effective measure for predicting NSSI outcomes.

The P3 amplitude were markedly smaller under negative stimuli or incongruent conditions, aligning with findings from previous studies (48). The P3 component is widely utilized as a biomarker in research on attention resource allocation associated with emotion and cognition (19). It is instrumental in assessing inhibitory processes, such as in the emotional signal stop task, where larger P3 amplitudes signify stronger inhibitory control (49). During conflict processing, suppressing responses to sad faces has been shown to reduce P3 amplitude (50). In the present study, the decreased P3 amplitude indicates that cognitive resources are heavily consumed by negative stimuli or conflicts during the Stroop task. Additionally, the P3 exhibited a gradient downward trend across the HG, RG, and AG, suggesting a progressive decline in the brain’s resource utilization efficiency in the NSSI group (51). A smaller P3 in adolescents implies less attentional allocation to task stimuli, likely due to emotional stimulus interference. Stimulus processing may divert attention, reducing task engagement. It may also signal impaired inhibitory control: limited cognitive control hampers focus by failing to suppress irrelevancies, consistent with poorer stimulus processing. Under neutral stimuli, AG participants demonstrated more severe P3 amplitude deficits than HG in congruent conditions. Similarly, RG participants displayed significant P3 amplitude deficits under negative stimuli, indicating notable cognitive control impairments compared to HG. These results highlight the importance of continued monitoring for RG participants, even though their NSSI behaviors are alleviated.

Findings support that adolescents with persistent NSSI have trouble mobilizing neural resources for inhibitory control when emotionally triggered. A larger N2 suggests conflict-monitoring struggles, while a smaller P3 indicates less attentional allocation and impaired inhibition. Together, these neural manifestations suggest that persistent self-injurers are more likely to resist the urge to injure and show more severe cognitive control deficits. Worth pointing out, Zhao et al. discovered that adolescents in the NSSI group displayed a smaller N2 and larger P3 in a two-choice oddball task with negative emotional stimuli (15). Similarly, Zhou et al. found that the NSSI group showed a larger P3 when exposed to self-injury cues in an oddball paradigm (52). These findings differ from the results of this study. Our findings might differ because of: 1) task variations that could elicit unique neural responses; 2) differences in sample age, gender, and NSSI severity; 3) our emphasis on outcome subgroups. Analyzing the aggravation group against others may uncover distinct NSSI-related neural mechanisms, enhancing field understanding.

Notably, AG participants showed an intermediate pattern under negative stimuli, with N2 and P3 amplitudes positioned between those of HG and RG. This may suggest that negative images become desensitized for AG participants due to frequent NSSI behaviors, resulting in reduced vigilance compared to RG (53). However, AG participants are “desensitized” to negative stimuli lacks direct evidence. In future research, measure skin conductance (a recognized arousal index) during negative stimulus presentation. Its changes, reflecting emotional responses, can provide objective data on AG participants’ reduced arousal, supporting the desensitization hypothesis. Alternatively, AG participants may experience more pronounced cognitive dysfunction, requiring additional resources to process negative stimuli, thereby impairing Stroop task performance (49, 54). Although the precise mechanisms remain unclear, these findings provide valuable insights into potential developmental trajectories.

In the RG and AG subgroups, a significant difference was observed in N2 amplitude but not in P3 amplitude, suggesting that AG participants experience greater difficulties with attention control over stimuli rather than resource allocation. This finding highlights impaired attention control as a central factor in the cognitive dysfunction associated with NSSI outcomes, corroborating earlier studies (55, 56). Clinicians can utilize the EEG as a neurophysiological marker to identify adolescents with ongoing NSSI behaviors, aiding in risk stratification to determine who needs more intensive intervention. The EEGnet model used in this study applies depthwise separable convolutional layers to optimize EEG feature extraction, encompassing spatial filtering and filter bank construction. Notably, the analysis demonstrated that N2 amplitudes elicited by neutral stimuli achieved the highest classification accuracy (92.31%) for predicting clinical outcomes of NSSI among the ERP components evaluated. The model’s high accuracy indicated a strong signal in the neural data that distinguishes outcome groups, supporting the ANOVA analysis results. These findings establish N2 amplitudes as reliable indicators for identifying clinical outcomes of NSSI, contributing to early detection and intervention strategies. This study suggests that N2 amplitude could serve as a biomarker for predicting NSSI outcomes, offering a potential avenue for developing interventions to reduce addiction risk. Improving attention control by addressing deficits in the N2 amplitude may effectively mitigate the risk of addiction in individuals with NSSI. The model demonstrated high accuracy, but this is a preliminary result from a small sample. Further research with larger groups is needed to confirm its generalizability. Given all that, cognitive control deficits are likely a central feature in adolescents with ongoing NSSI. Therefore, therapies aimed at enhancing executive function and emotion regulation, such as cognitive-behavioral therapies focused on impulse control and emotional processing, may aid in managing NSSI. Moreover, specialized cognitive training programs could be designed to tackle these deficits, though more studies are required to confirm their efficacy.

Behavioral data showed no accuracy difference between groups but slower RTs in the AG group, hinting at latent processing disparities despite similar correct-response performance. ERP results, however, revealed significant N2/P3 amplitude differences, indicating distinct task-related neural processing. EEG metrics (N2, P3) directly reflect neural activity during information processing. They can detect neural changes not shown in behavior. Although ERPs have shown differences between adolescents with and without NSSI, using EEG in clinical settings is difficult due to technical and resource constraints. However, these insights enhance our understanding of NSSI mechanisms and could guide future research, like larger studies or trials, to explore if therapeutic interventions improving cognitive control can reduce NSSI occurrence and severity. This study has several limitations. Sample size: small subgroup samples limit analysis. A larger sample would increase confidence and enable in-depth studies of comorbidities or sex differences, as the current one may miss nuanced effects. Population: all participants were Chinese adolescents from one region. Cultural factors impact NSSI. Results may not be global without further studies; future research should include diverse cultures. Follow-up: One-year follow-up may be insufficient for NSSI trajectories as behavior can change. A longer follow-up with larger samples to validate N2 as a stable biomarker and explore its utility in guiding interventions (e.g., cognitive training targeting attention control) is needed for long-term NSSI understanding. Potential confounds: depression, anxiety, medication use and comorbidities during follow-up may affect NSSI outcomes. Future studies should control these variables. Task specificity: the affective Stroop paradigm may not cover all inhibitory control. Different tasks/measures may vary results. Future studies should use multimodal approaches.

## Conclusions

This study employed a novel affective Stroop task to examine the neurocognitive mechanisms underlying different NSSI outcomes and to predict these outcomes using ERP components in adolescents. The findings demonstrated that NSSI is associated with abnormal cognitive processing, characterized by impaired attention control and resource allocation. Notably, N2 amplitudes emerged as reliable indicators for identifying clinical outcomes of NSSI. These results provide an objective foundation for identifying NSSI, predicting its developmental progression, and formulating early intervention strategies. To advance this research, we propose: validating these neural markers in a larger, diverse adolescent group with NSSI for better generalizability; conducting long-term studies to evaluate the stability and predictive value of ERP differences for NSSI outcomes; and exploring whether therapy can alter ERP differences to improve treatment understanding and interventions.

## Data Availability

The original contributions presented in the study are included in the article/supplementary material. Further inquiries can be directed to the corresponding author.
